# Pathophysiological Implication of Fetuin-A Glycoprotein in the Development of Metabolic Disorders: A Concise Review

**DOI:** 10.3390/jcm8122033

**Published:** 2019-11-21

**Authors:** Lynda Bourebaba, Krzysztof Marycz

**Affiliations:** 1 Department of Experimental Biology, Faculty of Biology and Animal Science, Wrocław University of Environmental and Life Sciences, Norwida 27B, 50-375 Wrocław, Poland; lynda.bourebaba@upwr.edu.pl; 2 International Institute of Translational Medicine, Jesionowa, 11, Malin, 55-114 Wisznia Mała, Poland; 3 Collegium Medicum, Institute of Medical Science, Cardinal Stefan Wyszyński University (UKSW), Wóycickiego 1/3, 01-938 Warsaw, Poland

**Keywords:** fetuin-A, hepatokine, metabolic disorders, insulin resistance, biomarker

## Abstract

Alpha 2-Heremans-Schmid glycoprotein, also known as fetuin-A (Fet-A), is a multifunctional plasma glycoprotein that has been identified in both animal and human beings. The protein is a hepatokine predominantly synthesized in the liver, which is considered as an important component of diverse normal and pathological processes, including bone metabolism regulation, vascular calcification, insulin resistance, and protease activity control. Epidemiological studies have already consistently demonstrated significant elevated circulating Fet-A in the course of obesity and related complications, such as type 2 diabetes mellitus, metabolic syndrome, and nonalcoholic fatty liver disorder (NAFLD). Moreover, Fet-A has been strongly correlated with many parameters related to metabolic homeostasis dysregulation, such as insulin sensitivity, glucose tolerance, circulating lipid levels (non-esterified free fatty acids and triglycerides), and circulating levels of both pro- and anti-inflammatory factors (C-reactive protein, tumor necrosis factor-α (TNF-α), and interleukin (IL)-6). Metabolic-interfering effects of Fet-A have thus been shown to highly exacerbate insulin resistance (IR) through blocking insulin-stimulated glucose transporter 4 (GLUT-4) translocation and protein kinase B (Akt) activation. Furthermore, the protein appeared to interfere with downstream phosphorylation events in insulin receptor and insulin receptor substrate signaling. The emerging importance of Fet-A for both diagnosis and therapeutics has therefore come to the attention of researchers and the pharmaceutical industry, in the prospect of developing new therapeutic strategies and diagnosis methods for metabolic disorders.

## 1. Introduction

Endocrine and metabolic diseases are considered nowadays to be among the most recurrent and clinically challenging coeval affections worldwide in human medicine, essentially due to a sharp decline in the lifestyle quality level in terms of surplus energy intake, increasing obesity, and sedentary life habits [1,2]. Metabolic syndrome (MetS), also described as syndrome X, is one of the most common metabolic disorders, which increases in prevalence as the population becomes more obese [3]. This condition mainly refers to a constellation of interconnected physiological, biochemical, clinical, and metabolic abnormalities including hypertension, central obesity, insulin resistance, and atherogenic dyslipidaemia (low high-density lipoprotein cholesterol, hypertriglyceridemia), which are closely associated with visceral adiposity [4,5]. Pathology in various target tissues that include the cardiovascular system, pancreas, and liver is usually relatively common in individuals suffering from MetS [6].

Insulin resistance (IR) is well recognized as being widely present in most types of metabolic disorders, such as obesity, dyslipidaemia, MetS, hypertension and atherosclerosis, nonalcoholic fatty liver disorder (NAFLD), type 2 diabetes mellitus (T2DM), and some cases of type 1 diabetes mellitus (T1DM). Indeed, IR, which refers to a complex pathological defect in insulin signalling pathways, resulting in inappropriate cellular response to insulin hormone in insulin-dependent tissues such as skeletal muscle, adipose, and liver tissues, has been proposed as a key underlying pathophysiological pathway in the onset of MetS [7,8,9]. Obesity-induced inflammatory cytokines have been closely related to IR, as many cytokines and inflammatory mediators, namely, tumor necrosis factor-α (TNF-α), monocyte chemotactic protein-1 (MCP-1), C-reactive protein (CRP), and interleukins, have been demonstrated to be significantly upregulated in the course of IR. On the other hand, adipocytes that are endowed with endocrine properties are prone to enable the release of inflammatory mediators such as leptin, TNF-α, and adiponectins, as well as plasminogen-activator inhibitor-1, angiotensinogen, and active steroid hormones, all of which participate strongly in the development of IR [10,11]. In addition, a number of studies have hypothesized that oxidative stress, a prominent feature of obesity, would be closely related to the chronic low-grade inflammation that characterizes MetS. Systemic oxidative stress could be thus be at least partly responsible for the dysregulated secretion of adipokines that contribute to the pathogenesis of MetS [12]. Cellular dysfunctions mediated by many soluble factors that may interact with each other, such as protein kinases and phosphatases, have also been highlighted in the course of IR. Impaired phosphorylation signaling pathway is known to trigger the decrease in insulin-stimulated glucose transporter 4 (GLUT-4) expression or translocation; resulting in alteration of glucose transport, reduced glycogen storage, and inhibited protein synthesis [13]. The hyperinsulinemia resulting state is thus capable of inducing serine/threonine phosphorylation of insulin receptor substrate (IRS), which is prone to promote IRS degradation and block the phosphorylation of tyrosine, which represents a key stage of continuing the remaining phosphorylation cascades of downstream targets, either phosphatidylinositide 3-kinases (PI3K) or a class of small GTPase (RAS) involved in PI3K/ protein kinase B (Akt)/ Forkhead box transcription factors of the class O (FOXO1) signaling pathways [14].

Fetuin-A (Fet-A), as known in human as α2-Heremans-Schmid glycoprotein (AHSG), is a member of the protease inhibitors cystatin superfamily, which refers to a major serum glycoprotein mainly secreted by liver tissue. Studies have demonstrated that Fet-A protein is involved in important physiological cellular functions, such as cellular protein and fatty acid metabolism, regulation of acute inflammatory responses, regulation of bone mineralization and calcified matrix metabolism, neutrophil degranulation, lymphocyte recruitment, and thyroid hormones and calcium ion homeostasis, among others [15]. On the other hand, the glycoprotein has been recently proposed as a molecular link between both obesity, NAFLD, IR, and MetS, as it has been associated with inhibition of the insulin receptor activity, leading to a breakdown in insulin cascades pathways [16]. Application of advanced techniques in biochemical, immunochemical, and molecular genetics has profoundly enriched the knowledge regarding Fet-A protein during the last few years, generating relevant outcomes concerning its value as a biomarker in the diagnosis and therapy of human diseases [17].

This review will therefore be dedicated, firstly, to the summary of current available data relating to the different physiological roles exerted by fetuin-A protein on the various metabolic pathways, as well as to the discussion of its involvement in the initiation, promotion, and development of various metabolic disorders at the molecular level.

## 2. Role of Fetuin-A in Physiological Processes

Fetuin-A (AHSG) is a 52 kDa glycoprotein that is predominantly synthesized by hepatocytes and subsequently excreted in the bloodstream, and is therefore commonly considered as a hepatokine. The first report relating to Fet-A described high amounts of the protein in calf fetal serum, and demonstrated that its concentration decreases with the age of the animal. Claimed as the most abundant globular plasma protein in calf serum, the protein was consequently named as “Fetuin”, and has been later renamed as Fetuin-A, which refers to a phosphorylated liver glycoprotein that is systemically distributed via the bloodstream [18,19]. Human fetuin is more exactly known as α2-Heremans-Schmid glycoprotein, which is encoded by the *Ahsg* gene mapped to the 3q27 region of human chromosome 3, and transcribed as a single messenger RNA (mRNA). The α2 value was attributed due to the comigration of fetuin with the α2-globulin fraction of serum proteins during electrophoresis on cellulose acetate. Furthermore, the term “Heremans-Schmid glycoprotein” was dedicated to the researchers Heremans and Bürgi, and Schmid, who identified for the first time the human homologue of fetuin in 1960 and 1961, respectively [20,21]. Fet-A belongs to the cystatin superfamily and is composed of two subunits, a heavy A chain consisting of about 282 amino acids, and a light B containing 12 amino acids. The two chains are connected to each other by half-cystine residues of their amino acid sequences, which are subsequently organized into a loop structure [22]. 

**Figure 1:**
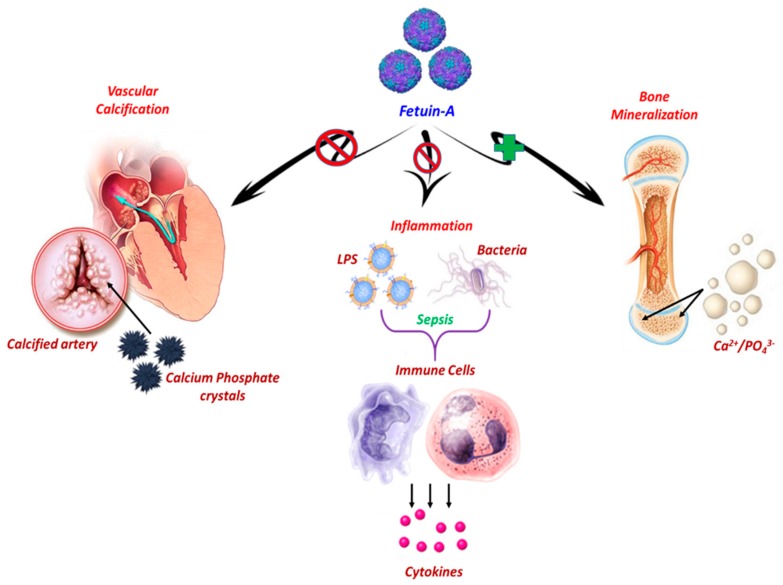
Graphical representation of main physiological roles of hepatic-secreted Fetuin-A. Under physiological conditions, Fetuin-A (Fet-A) enhances the absorption and fixation of essential minerals, calcium, and phosphate in the form of hydroxyapatite substituted with a carbonate. Moreover, Fet-A acts as a carrier of insoluble phosphate and calcium, and forms stable mineral complexes, more soluble in the bloodstream, preventing the precipitation of calcium salts through the mediation of their clearance, and the consequent occurrence of vascular calcifications. Fet-A functions as a negative regulator of the innate immune response by inhibiting Lipopolysaccharide (LPS)- or Interferon (IFN)-γ-induced High mobility group box 1 protein (HMGB1) release in macrophages in response to lethal endotoxemia or sepsis.

Physiological plasma concentrations of Fet-A generally range from 0.4 to 0.8 mg/mL in humans to several milligrams per milliliter in fetal calf serum; as a result, the use of bovine serum for cell cultures, for example, inevitably exposes the cells to large proportions of Fet-A, with this latter being able to promote cell adhesion, proliferation, and differentiation [18,23,24]. Like albumin, Fet-A is widely distributed in the extracellular space of virtually all vascularized tissues, and is therefore completely absent from avascular tissues such as cartilage. Mineralized bone, as well as dentin, represent the tissues in which Fet-A is considered as one of the most abundant non-collagenous proteins with a high affinity for the apatite mineral, which makes it probably a crucial factor for mineral metabolism [19,25]. Due to a rather large expression and the significant capacity for molecular interactions with several different ligands, it was assumed that Fet-A could mainly exert support and scavenging functions similarly to albumin, and would play a key role in several physiological processes (Figure 1) [19].

### 2.1. Fetuin-A and Bone Metabolism

Biomineralization is defined as the entire process by which living organisms elaborate and produce highly resistant and specific hard tissues, which are essential for many vital functions such as movement and support (bone), nutrition (teeth), as well as calcium, phosphate, and other electrolyte metabolism. The fundamental principle of biomineralization lies in the deposition of various mineral ions, of which the most commonly involved are calcium, silicon, iron, barium, and strontium, in the context of organic substrates, in order to achieve sufficient tissue hardness. It also refers to all of the metabolic reactions involved in the formation of these tissues [26,27]. Bone represents the most abundant calcified tissue in vertebrates. Its calcification is strictly dependent on two main parameters, namely, metabolic function of bone cells as well as properties of the bone matrix [27]. Bone matrix, which provides the mechanical stabilization of bone tissue, essentially requires mineralization based on calcium and phosphate deposition in the form of hydroxyapatite substituted with a carbonate (carbonate apatite). In addition to collagen type I, which is the main protein of this matrix, other non-collagenous bone proteins are also found, the most prominent of which are osteocalcin, osteopontin, and Fet-A, which play a pivotal role in the various mineralization metabolic reactions [28,29]. The presence of fetuin-A in mineralized bone tissue, as well as in calcified lesions of the vascular system (i.e., atherosclerotic) in significant proportions, clearly indicates that the glycoprotein exhibits a fairly high affinity for mineralized tissues [30,31]. A number of substantial data generated over the past few years clearly indicate that fetuin-A primarily intervenes in bone metabolism by binding calcium and transforming growth factor beta (TGF-β). Fet-A regulates bone mineralization by inhibiting osteogenesis through its ability to bind both TGF-β and bone morphogenetic proteins (BMP). During calcification, the apatite particles must be deposited in the intrafibrillar space of the collagen type I network. Fet-A, being unable to penetrate into this space because of its size, is responsible for the limitation of the mineral deposit outside the collagen matrix, and thus manages to restrict the mineralization to the intrafibrillar space only. Aside from this, osteocalcin, which is synthetized by bone cells, is small enough to integrate the collagen network (10.9 kDa). Its activity, which consists essentially in mediating the deposition of small apatite crystals in a controlled manner, is then mediated through a vitamin K2-dependent gamma-carboxylation of glutamic acid residues, which ensure the proper bone mineralization [32,33]. The exact mechanism by which Fet-A manages to regulate and maintain bone tissue homeostasis, lies mainly in its antagonizing action with respect to the respective effects of TGF-β and BMP isoforms in a competitive manner. The glycoprotein is thus prone to create an inhibitory complex with one of the two factors, and to interfere in their binding to the extracellular domain of TGF-β receptor (TβRII), due to the presence of a common major cytokine binding site in Fet-A and TβRII [34]. Apart from its prominent role in the control of osteogenesis, fetuin-A is also a physiological inhibitor of bone calcification. It has thus been previously demonstrated that the protein could significantly inhibit the formation of apatite in an in vitro calcification model composed of glycerophoshate-induced rat calvaria osteoblasts cultures. In addition, these inhibitory effects attributed to fetuin-A have also been successfully demonstrated following a salt precipitation measurement. Interestingly, inhibitory effect of fetuin on apatite formation was abolished following subsequent chemical blocking of protein-carboxylate groups using glycine ethyl ester, highlighting that aspartic and glutamic acid residues would be closely involved in calcium binding, and that the inhibitory effect of Fet-A is probably mediated by acidic amino acids clustering in cystatin-like domain D1 [35].

### 2.2. Fetuin-A and Vascular Calcification

As an important mediator involved in the regulation of calcium metabolism, Fet-A is known to strongly reduce and suppress systemic calcification, as the decrease in serum fetuin concentration has been closely associated with exacerbation of soft tissue calcifications, as well as stiffening and calcification of the arteries. Fet-A acts as a carrier of phosphate and calcium when they are in their insoluble form. The remaining mineral complexes become more stable and soluble in the bloodstream, and thus prevent the precipitation of calcium salts through the mediation of their clearance, as well as the consequential occurrence of vascular calcifications [36,37,38]. Interaction of Fet-A with calcium and phosphate surplus allows the formation of calciprotein monomers (CPP). The CPP thus tend to accumulate in the form of higher aggregates, giving rise to amorphous calcium phosphate-rich nanoparticles, containing about 18% minerals, 80% fetuin-A, and 2% matrix Gla protein (MGP), usually reaching a particle size varying between 30 and 150 nm [39]. These aggregates are subsequently eliminated, either via the scavenger-receptor pathways of the reticuloendothelial system, which prevents their deposition and thus calcification, or by spontaneous rearrangement into secondary or mature CPP after addition of albumin and proteins acids [40]. The formed secondary CPP, which exhibit larger sizes, are considered as more stable and less cytotoxic than their primary precursors. Being coated with a layer composed of Fet-A and serum albumin, they can be easily removed and cleared from the circulation. Molecular modelling studies have concluded that the inhibitory potential on phosphate and calcium deposition is mainly mediated by the group of acidic residues on Fet-A domain 1 β-sheet, similar to cystatin, the amino-terminal end [41,42]. In this regard, the study of Fet-A-deficient mice demonstrated that animals were more prone to the development of extensive soft tissue calcification, as compared to mice expressing normal Fet-A levels, thus confirming that the protein has a crucial role in the mechanisms of systemic inhibition of extraosseous mineralization [37,43]. Moreover, certain in vitro and in vivo investigations have pointed out the evidence that Fet-A is a central factor for the absorption of minerals by vascular smooth muscle cells (VSMCs), which could then limit calcification by reducing cleavage of caspases and apoptosis, which are considered as key events during vascular calcification [44]. In humans, Fet-A deficiency has been strongly linked to increased arterial calcification scores and significant higher mortality incidences. Low circulating Fet-A concentrations have also been evidenced during progressive aortic stiffening and calcification in the course of some kidneys pathologies [45,46,47].

### 2.3. Fetuin-A and Inflammatory Response

Fetuin-A has been previously proposed as a potent anti-acute phase protein in sepsis, however, many suggest that it plays a much more complex role in inflammatory processes and that it would be a stimulatory or suppressive protein in the acute phase according to the respective stimuli. During inflammation, pro-inflammatory cytokines involved in the early phase, such as TNF-alpha, interleukin (IL)-1, IL-6, and IFN-γ, are prone to suppressing the expression of Fet-A, and thus to exacerbating the inflammatory response and the resulting excessive accumulation of late mediators, with essentially high mobility B1 protein, (HMGB1); the presence of these latter in abundance will consequently restore the physiological liver secretion of Fet-A during the last stages of sepsis [48,49]. Fet-A was also reported to closely interact with immune cells, and to exhibit potent opsonin functionality to cationic spermine, making it a crucial element for regulating the innate immune response [50]. Treatment of LPS-stimulated primary human monocytes with spermine suppresses the synthesis of pro-inflammatory cytokines such as TNF-α, 1L-l, 1L-6, macrophage inflammatory protein (M1P)-lα, and M1P-lβ in a dose-dependent manner [51]. Moreover, the mechanism by which spermine suppresses cytokine release including TNF-α was reported to be post-transcriptional, as application of spermine to LPS-induced macrophage cultures did not noticeably modify the levels of TNF-α mRNA. Therewith, exogeneous spermine failed to suppress TNF-α secretion in partially starved macrophage cultures (human peripheral blood monocytes or murine macrophages cells) when culture media were depleted with Fet-A, evidencing that fetuin is an important requirement for macrophage responses to spermine. Furthermore, it has been demonstrated that TNF-α abrogating effects of spermine can be completely restored following supplementation of highly purified exogenous fetuin, but not by other serum glycoproteins [52,53].

Stimulation of cultured macrophages with 100 ng/mL of LPS was demonstrated as significantly reducng the levels of cellular Fet-A by approximately 40%. This effect was afterwards shown to be reversed by 30–50% after culture-supplementation with 100 µg/mL Fet-A, which was evidenced to be predominantly localized in Microtubule-associated protein 1A/1B-light chain 3 (LC3)-containing cytoplasmic vesicles—possibly autophagosomes or amphisomes after macrophage internalization—confirming the theory that macrophages could respond positively to exogenous fetuin stimulation. Higher concentration of purified Fet-A (3.5 mg/mL) was also found to almost totally suppress IFN-γ- or endotoxin-induced production of interleukin 1β (IL-1β), HMGB1, nitric oxide (NO), and tumor necrosis factor-alpha (TNF-a), whereas that of IL-6 remained unaffected in cultured macrophages, suggesting that fetuin-A is indeed a potent anti-inflammatory acute phase protein (APP) [48,49,54]. Fet-A modulation on hydroxyapatite (HA) crystal-stimulated human inflammatory cells was also clearly established. Indeed, accumulation of basic calcium phosphate (BCP) crystals is usually associated with the occurrence of acute articular and periarticular inflammation, which can be assimilated to acute gout. Treatment with fetuin protein successfully inhibits HA-induced neutrophil superoxide release, and partially restores inhibitory activity to HA-adsorbed serum (60%). Fet-A is also able to bind to BCP crystal and octacalcium phosphate in vitro. The presence of N-linked and O-linked oligosaccharide chains, whose terminal sugar residues are rich in sialic acid moieties in Fet-A structure, gives it the carrier function of several biologically active molecules such as calcium ions, and thus the binding properties of Fet-A may potentially modulate the inflammatory effects of BCP crystals [50,55].

## 3. Fetuin-A in Metabolic and Cardiovascular Disorders

The metabolic syndrome (MetS) is nowadays a major and growing public health and clinical challenge worldwide, encompassing many underlying dysfunctional mechanisms such as insulin resistance, inflammation, hormonal changes, and physical activity [56]. 

Obesity-associated lipolysis triggers the generation and release of free fatty acids into the bloodstream in excess, subsequently increasing chronic subclinical inflammation, thereby exacerbating pre-existing insulin resistance in various targeted tissues [57,58]. Non-alcoholic fatty liver disease (NAFLD), which counts as one of the most common metabolic syndrome-related hepatic manifestations, is part of a constellation of chronic liver diseases, ranging from simple steatosis, or non-alcoholic steatohepatitis (NASH), to liver cirrhosis [59,60].

**Figure 2:**
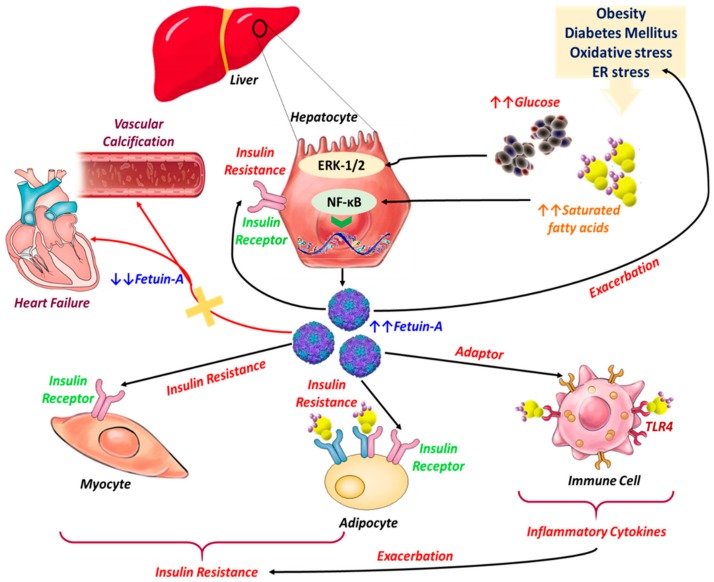
Pathophysiological implications of Fet-A in the course of metabolic and cardiovascular diseases. Excessive release of Free fatty acids (FFAs) and overaccumulation of glucose in the bloodstream stimulate the biosynthesis of Fetuin-A from hepatocytes through activation of both Nuclear factor kappa B (NF-κB) and ERK 1/2 pathways. Fet-A acts then as an insulin signaling pathway inhibitor by modulating the kinase reaction initiated by insulin on insulin-receptor tyrosine kinase autophosphorylation; insulin-sensitive tissues become less responsive to insulin, triggering insulin resistance. Meanwhile, Fet-A functions as an adaptor between FFA and Toll like receptor 4 (TLR4) signaling in lipid-induced inflammation, and TLR4 signaling leads to the activation of NF-κB and Activator protein 1 (AP-1), which can then upregulate the transcription of inflammatory genes, resulting in the production of inflammatory cytokines that can lead to insulin resistance. On the other hand, decreased Fet-A synthesis is strongly correlated with excessive vascular calcification and general heart failure, as the Fet-A-stabilization effect toward insoluble calcium and phosphate crystals is no longer properly achieved.

Organokines are known to be strongly involved in cellular metabolism through autocrine, paracrine, and endocrine activity. Recent experiments have demonstrated that liver tissue plays a key role in the control and maintenance of whole-body energy homeostasis by regulating glucose and lipid metabolism through the secretion of several hepatokines, among of them Fet-A protein, thereby evidencing that liver and its related hepatokines are largely involved in the development of certain metabolic disorders (Figure 2) [61,62,63]. Cardiovascular disease (CVD), which refers to conditions affecting the cardiovascular system, including coronary artery disease, angina, and heart failure, has become for the most part one of the leading causes of mortality worldwide; hence, more individuals die from CVD than from any other disorder each year [64]. Several studies have established that coronary calcification determination may facilitate cardiovascular risk prediction independently of other conventional risk factors. As Fet-A is one of the most important inhibitors of calcification, it has recently gained more attention as a valuable factor linked with cardiovascular mortality [65]. Circulating fetuin-A levels are thereby found to be strongly increased in the course of obesity, metabolic syndrome, and type 2 diabetes, and are correlated with hepatic steatosis and CVD in humans [66].

### 3.1. Fetuin-A, Insulin Resistance, and Diabetes Mellitus

The first investigation that reported on the implication of Fet-A in insulin-signaling pathway was conducted by Auberger and colleagues [67]. They isolated and characterized a 63 kDa glycoprotein (pp63) secreted from adult rat hepatocytes that exhibited an unusual property for a secretory protein, being phosphorylated, subsequently identifying it as the homologue of human Fet-A after rat liver complementary DNA (cDNA) isolation and analysis. They demonstrated through their investigation that purified pp63 protein in a dose-dependent manner inhibited the kinase reaction initiated by insulin on insulin-receptor tyrosine kinase autophosphorylation in a cell-free system [67]. Subsequently, several other groups also evidenced that both purified rat fetuin-A (pp63) and bovine fetuin-A were prone to suppress insulin receptor tyrosine kinase in vitro. Later, serum-purified human Fet-A was also proven to substantially disrupt insulin-stimulated phosphorylation of IR and IR substrate-1 (IRS-1) [68,69,70]. In another study, Fet-A significantly reduced both basal and insulin stimulated phosphorylation of E26 transformation-specific (ETS) Like-1 protein (Elk-1) signaling, a transcription factor phosphorylated and activated by mitogen-activated protein kinase and other related upstream kinases, without affecting insulin-stimulated translocation of GLUT-4 or glucose transport, in transfected rat adipocytes overexpressing human alpha2-HSG cultures [71].

The exact role of Fet-A in insulin receptor (IR) signaling in genetically modified rat 1 fibroblasts overexpressing the human IR (HIRc B) has been tentatively established. The inhibitory effects of recombinant alpha2-HSGbac (at 1.8 µM) on the insulin-stimulated phosphorylation of IR and IRS-1 have been demonstrated and quantified at over 80%, and was mainly attributed to direct interaction between fetuin-A and IR after IR was co-immunoprecipitated with fetuin-A in response to insulin. Meanwhile, Fet-A did not modulate epidermal growth factor (EGF) or insulin-like growth factor I-induced cognate receptor autophosphorylation, suggesting a relative specificity of Fet-A for IR; however, the specific site of Fet-A and IR interaction had not been highlighted at that point. Further attempts to structurally characterize the Fet-A interaction with IR have been studied through the trypsinisation of IR autophosphorylation in HIRc B cells. This resulted in proteolysis of the IR α-chain and constitutive activation of insulin receptor tyrosine kinase activity (IR-TKA). Fet-A subsequently totally inhibited the trypsin-activated IR autophosphorylation and TKA in vitro, suggesting that IR modulation cannot be mediated by interaction of Fet-A with the proximal 576 amino acid residues of the IR α-subunit, and indicating that the glycoprotein may not compete with insulin for binding to IR [72].

Afterwards, researchers established that Fet-A is more likely to bind to tandem fibronectin type 3 (Fn3) domains, present within the 194 amino acid residue extracellular moiety of the insulin receptor β-subunit. The evidence of a molecular cooperativity then clarified that the two proteins, insulin and fetuin, exhibit an affinity for the same insulin receptor ectodomain, with the former activating the receptor’s intrinsic tyrosine kinase (TK) activity through its binding to the α-subunit, creating a conformational change that promotes the binding of the latter to the β-subunit, resulting in the receptor inactivation [73].

To the extent that fetuin-A has been formally recognized as being directly involved in the development of insulin resistance, in both humans and animals, researchers have extensively recorded the emergence of insulin resistance and obesity concomitantly to higher fetuin-A levels. Insulin resistance and obesity are the main major risk factors for type 2 diabetes. Therefore, several studies have focused on highlighting a possible link between Fet-A levels and risk of type 2 diabetes [74,75,76]. Recent meta-analyses of cohort studies showed that patients suffering from type 2 diabetes have been diagnosed as exhibiting higher fetuin-A levels compared to non-diabetic individuals; in addition, non-diabetics with high fetuin levels showed a higher risk (23% to 24%) of developing the condition [77,78,79].

Several underlying molecular mechanisms, primarily insulin resistance, may contribute to the development of Fet-A-associated diabetes. The increase in Fet-A concentrations already showed positive correlation with development of obesity both in human and animal models, whereas weight loss has been observed to be proportionally linked to reduced levels of Fet-A [80]. Fetuin-A is additionally responsible for promoting lipid-induced insulin resistance by the enhancement of free fatty acids binding to toll-like receptor 4 (TLR4) through an endogenous ligand function mediated by its terminal galactoside moiety, which is able to directly bind the Leu100–Gly123 and Thr493–Thr516 residues in TLR4, leading to adipose tissue inflammation and subsequent onset of insulin resistance [81]. In adipose tissue, Fet-A has been shown to downregulate the expression of adiponectin, thereby suppressing its anti-inflammatory and insulin-sensitizing properties [82].

### 3.2. Fetuin-A in the Course of Obesity

A wide range of experimental and clinical data constitute a body of proof concerning the obvious role of fetuin-A in obesity. Implication of inflammation in adipose tissue was substantiated experimentally in obese diabetic mice—Fet-A reduced adiponectin expression in a lipid-induced inflamed adipocytes model, leading to a sharp decrease in peripherical insulin sensitivity related to dysregulated lipid metabolism, as adiponectin is known to play crucial physiological roles in diabetes and atherosclerosis. Moreover, treatment of 3T3L1 adipocyte with Fet-A, resulted in a stimulation of Wnt3a expression in a dose-dependent manner. As a consequence of Wnt3a overexpression mediated by Fet-A, production of Peroxisome proliferator-activated receptor gamma (PPARγ) and adiponectin was strongly reduced. This was consistently confirmed when Fet-A was demonstrated to be ineffective in decreasing PPARγ and adiponectin levels in Wnt3a gene knocked-out 3T3L1 adipocytes. These observations indicated that Fet-A may mediate its inhibitory effect on adiponectin through Wnt3a/PPARγ pathway [83].

Fetuin-A and adiponectin are believed to work in concert to regulate insulin resistance. Both proteins are encoded by respective genes that are mapped to 3q27 in the human genome, a well-established diabetes and metabolic syndrome susceptibility locus. Circulating concentrations of both proteins are likely associated with key components of MetS, remaining, however, in opposite directions. Importantly, body mass index and hypertriglyceridemia are for example closely correlated with significant high levels of Fet-A, although they are related to lower adiponectin concentrations [84]. Cultured human adipocytes no longer express the adiponectin gene after being exposed to the Fet-A glycoprotein, whereas treatment of wildtype mice with Fet-A markedly lowered serum adiponectin levels. Thus, it was assumed that modulating effects of Fet-A are strictly adiponectin-specific, owing to the fact that none of the other circulating adipokines, namely, leptin and resistin, were affected by Fet-A at both mRNA and protein levels. Overall, the different collected data clearly confirm that liver-secreted protein fetuin-A inhibits and suppresses synthesis of adiponectin in adipose tissue, and therefore support the evidence that higher fetuin-A hepatokine and lower adiponectin may strongly contribute to obesity-induced insulin resistance and the development of diabetes [82].

Accumulation of lipids in the adipose tissue environment has been proposed as a triggering factor of fetuin-A abnormal secretion. Adipose tissue samples obtained from obese diabetic *db/db* mice, high fat diet-fed mice, and obese diabetic patients exhibited significantly elevated Fet-A levels as compared to their controls; however, partially hepatectomized high fat diet mice did not show a noticeable alteration, indicating adipose tissue to be the main source of this aberrant secretion. Free fatty acid-mediated Fet-A expression at both gene and protein levels in adipose tissue has been observed in another model of obese diabetic mice. The overproduced protein was also shown to act as a chemoattractant for macrophage recruitment and migration into adipose tissue and their subsequent polarization to M1 pro-inflammatory secreting subtype [85]. Further analysis of white fat explants of both visceral and subcutaneous origin, and at different physiological and nutritional status, including anorexia and obesity, evidenced noticeable differences in Fet-A secretion, demonstrating higher expression levels in visceral adipose tissue, correlated with the occurrence of obesity that was easily regulated by exercise and fasting, unlike adipose tissue explants sampled from anorectic animals that exhibited markedly reduced Fet-A glycoprotein secretion. On the other hand, subcutaneous secretion of an active, phosphorylated form of Fet-A appeared to be intensively incremented in obesity, contributing to the onset of insulin resistance [86].

Meta-regression analysis demonstrated that dysglycaemic and overweight/obese adult and old patients exhibited lowered circulating Fet-A after supervised exercise interventions in both aerobic and resistance exercise at vigorous or moderate intensity, with a volume of 60 min/session and a minimum frequency of four to seven sessions/week. This was supported by the fact that physical exercise significantly modulates adipokine levels, inflammatory cytokine, and glycaemic control in youths and patients with type 2 diabetes [87,88,89]. Other experiments showed that a 12-week caloric restriction strategy implementation in overweight rats and humans with type 2 diabetes respectively resulted in consequent reduction in hepatic expression and circulating levels of Fet-A, as well as insulin resistance alleviation. In addition, observed changes in Fet-A levels during caloric restriction protocol were significantly associated with changes in adiponectin levels, which is a pivotal anti-inflammatory adipokine. Both circulating atherogenic apolipoprotein B (apoB) and low-density lipoprotein (LDL)-cholesterol levels were also shown to be significantly lowered after 12 weeks of caloric restriction, which positively correlated with decreased circulating Fet-A [90].

Current available data have provided substantial evidence that body mass index (BMI), waist-to-hip ratio, a body shape index (ABSI), triglycerides (TG) levels, fasting blood sugar (FBS) levels, HbA1c levels, C-peptide levels, and homeostasis model assessment (HOMA)-IR index can be efficiently decreased following bariatric surgery, including either gastric bypass (GB) and sleeve gastrectomy (SG), supporting the important role of that these kinds of surgical strategies have in maintaining long-term weight loss and improving glycaemic control [91,92,93]. The investigation of fetuin-A levels’ correlation with bariatric surgery demonstrated inconsistent and divergent outcomes. Indeed, concentration of Fet-A has been shown to increase at 4 months, remain unchanged at 6 months, decrease at 12 months, or decrease at 16 months in morbidly obese individuals after GB. Furthermore, circulating Fet-A has also been demonstrated to be either unchanged at 6 months or decreased at 12 months in morbidly obese individuals after SG [80,94,95]. Serum concentrations of Fet-A have additionally been reported to be positively correlated with BMI in diabetic and obese patients, highlighting positively correlated changes in Fet-A levels with those in BMI and waist-to-hip ratio 12 months after GB surgery [96]. Therewith, circulating serum Fet-A levels independently predicted serum triglycerides before as well as during the first 6 months following bariatric surgery [97].

### 3.3. Fetuin-A and Its Relation to NAFLD

The involvement of fetuin-A in pathological liver fat accumulation was studied in various investigations, as fatty liver disease is closely related to insulin resistance and dyslipidemia [98]. Liver tissue is implicated on the regulation of cellular metabolism, mainly through the synthesis and release of specific circulating proteins, known as hepatokines, which are especially engaged in the regulation and maintenance of clearance as well as glucose and lipid metabolism. Previous reports stated that overproduction of such kinds of glycoproteins in hepatocytes may take place in the course of steatosis and inflammation [76].

Differential display gene expression analysis of rats fed high- or low-fat diets demonstrated divergent expression levels of Fet-A mRNA in the livers of rats fed a high-fat diet, but not in groups that received a low-fat diet [99]. Furthermore, Fet-A hepatokine transcription was shown to be upregulated in mouse livers that had high-fat diet-induced overaccumulation of fat [76]. Relevant overexpression and activation of NF-κB in rat hepatocytes has been also revealed. To consolidate these results, NF-κB-knockout HepG2 cells were cultured in the presence of palmitate, which failed to stimulate Fet-A secretion, whereas induced expression of NF-κB was effective in consistent triggering Fet-A secretion, even in the absence of palmitate [100]. Fet-A has been extensively linked to lipid-induced insulin resistance in humans. Hepatic production of the glycoprotein has been shown to be triggered via NF-κB and growth-factor responsive mitogen-activated protein kinase (ERK 1/2) signaling pathway activation through high levels of saturated fatty acids and increased blood glucose concentration, respectively [101].

Much research has pointed out the significant increase of Fet-A synthesis in patients suffering from hepatic steatosis and morphologically confirmed NAFLD conditions, which were subsequently closely related to ectopic fat accumulation in the liver. Haukeland et al. [102] established that liver-produced Fet-A expression at the protein level was positively related to main lipid and glucose metabolism enzymes such carnitine palmitoyl-transferase 1 (CPT-1), sterol regulatory element-binding protein 1c (SREBP1c) and fatty acid synthase (FAS), phosphoenol pyruvate kinase 1 (PEPCK-1), and glucose-6- phosphatase (Glu-6-P), whereas no obvious differences in Fet-A mRNA transcript levels in liver tissue of NAFLD patients were recorded when compared with control cohorts [102]. Other experiments highlighted the important role of Fet-A in palmitate-induced hepatic lipid accumulation in hepatocytes. The hepatokine critically mediated accumulation of triacylglycerol in hepatocytes, activated SREBP-1c expression, and stimulated inflammatory cytokines in monocytes and adipocytes as a consequence of adiponectin suppression [82]. Moreover, addition of adiponectin was successful in regulating liver Fet-A expression through adenosin monophosphate-activated protein kinase (AMPK)-mediated reduction of NF-κB activity, which was blocked by AMPK small interfering RNA (siRNA) or AMPK inhibitor [103]. Fet-A mRNA expression was found to be higher in the nonalcoholic steatohepatitis (NASH) population, as compared to patients with simple fatty liver [94]. Moreover, treatment of individuals with NAFLD with metformin successfully reduced serum Fet-A levels, although no histological improvement was found in the same patients [102,104]. Body weight loss strategy in obese NAFLD patients led to hepatic fat content normalization, which consequently resulted in diminished Fet-A serum concentrations [16]. Positive regulation of serum concentration of this hepatokine has also been reached after a short-term, seven-day physical exercise trial, despite no visible effect on body weight or hepatic triglyceride content being recorded [105].

### 3.4. Fetuin-A in Cardiovascular Diseases

Atherosclerotic cardiovascular disease (CVD), a major, plaque-forming degenerative arterial affection, is considered as being among the leading causes of morbidity and mortality in cardiovascular death worldwide [106]. Besides the many typical Framingham risk factors, such as hypertension, dyslipidaemia, and diabetes mellitus, which are being considered to be largely responsible for the excessive CVD burden, more recent studies have shown that other less conventional risk factors, including inflammation, oxidative stress, and vascular calcification, could also contribute to the occurrence of the condition [107].

Recently, clinical implications of fetuin-A in the course of CVD have been pointed out. Despite the fact that Fet-A exhibits atherogenic effects in metabolic syndrome, the protein has been described as being importantly involved in the protection against atherosclerotic calcification [37].

Association of fetuin-A with atherosclerotic disease has thus been the subject of many investigations; patients suffering from symptomatic CVD exhibited increasing concentrations of Fet-A in coronary artery disease (CAD), in opposition to peripheral artery disease (PAD); moreover, Fet-A appeared to correlate positively with fatty liver disease in CAD, in contrast to PAD, thereby supporting the hypothesis that Fet-A might play distinct roles during atherosclerosis depending on the affected artery arterial [108].

On the other hand, Fet-A was inversely correlated with lower extremity arterial calcification in patients with simultaneous type 2 diabetes and PAD [109]. Epidemiological investigation of over 2000 clinical cases highlighted that fetuin-A hepatokine was more likely to be related to impaired glucose metabolism and completely dissociated from CVD [110]. Similar trends were observed in patients affected by obstructive sleep apnea syndrome, where Fet-A appeared to be inversely linked with subclinical carotid atherosclerosis [111].

To another extent, owing to the fact that fetuin-A is a circulating negative acute-phase glycoprotein that strongly inhibits ectopic Ca/PO_4_ ion precipitation and vascular calcification, it is assumed that its deficiency may closely contribute to cardiovascular dysfunction [112].

Fet-A serum levels were previously determined in almost 312 prevalent haemodialyzed patients, and were shown to be significantly decreased in comparison to normal or high-normal Fet-A level patients. Thus, decreased Fet-A concentrations may link inflammation, accelerated atherosclerosis, and CVD in uremic subjects and could likely explain in part the high prevalence of vascular calcification in this population [45].

Other investigations have evidenced significantly lowered median fetuin-A level in end-stage renal disease (ESRD) patients, with obvious inflammatory states, characterized by both IL-6 and high-sensitivity C-reactive protein (hs-CRP) overexpression, with concomitant interleukin-1β-triggered down-regulation of Fet-A hepatic mRNA, indicating that Fet-A serves as a negative acute-phase reactant [45,113].

Otherwise, differential involvement of Fet-A to CVD mortality risk according to diabetic status has been clearly established. Low levels of circulating Fet-A were shown to correlate with almost 76% higher risk of CVD death in non-diabetic subjects, and were instead associated with only 57% lower risk of CVD mortality in patients suffering from diabetes. Those significant associations were demonstrated to be completely independent from usual CVD risk factors, insulin resistance, and measures of liver and kidney function. These findings seem to consolidate the hypothesis that fetuin-A hepatokine protects against vascular calcification, but promotes on the other hand insulin resistance and metabolic dysregulation, suggesting that the balance between these two functions may be conditioned by metabolic milieu or prior pathophysiological processes [114].

## 4. Concluding Remarks

To date, a number of in vitro and in vivo experimental studies on different animal models, as well as clinical trials involving humans, have clearly demonstrated that fetuin-A is a pivotal glycoprotein in the regulation, maintenance, and even modulation of various metabolic pathways, essentially involving glucose, lipids, and minerals. Apart from its important physiological activities, it has been reported that fetuin-A may positively correlate with markers of early atherosclerosis, metabolic syndrome, obesity, IR, NAFLD, and low-grade inflammation of adipose tissue. High circulating fetuin-A level was also shown to constitute a strong predictor of incident T2DM, although the exact underlying molecular mechanisms remain poorly understood. In that prospect, there is a body of evidence that fetuin-A could serve as new molecular target in the elucidation of complex pathophysiological pathways involved in the onset of metabolic disorders, and could also be used as a useful marker in clinical practice in the future for the early diagnosis of these conditions.
